# Application and demonstration of meso-activity exposure factors to advance estimates of incidental soil ingestion among agricultural workers

**DOI:** 10.1038/s41370-024-00671-0

**Published:** 2024-05-17

**Authors:** Sara N. Lupolt, Brent F. Kim, Jacqueline Agnew, Gurumurthy Ramachandran, Thomas A. Burke, Ryan David Kennedy, Keeve E. Nachman

**Affiliations:** 1 Department of Environmental Health & Engineering, Johns Hopkins Bloomberg School of Public Health, Baltimore, MD USA; 2 Johns Hopkins Center for a Livable Future, Johns Hopkins Bloomberg School of Public Health, Baltimore, MD USA; 3 Risk Sciences and Public Policy Institute, Johns Hopkins Bloomberg School of Public Health, Baltimore, MD USA; 4 Department of Health Policy and Management, Johns Hopkins Bloomberg School of Public Health, Baltimore, MD USA; 5 Department of Health Behavior and Society, Johns Hopkins Bloomberg School of Public Health, Baltimore, MD USA

**Keywords:** Exposure modeling, Vulnerable occupations, Workplace exposures, Dermal exposure, Pesticides

## Abstract

**Background:**

Soil is an understudied and underregulated pathway of chemical exposure, particularly for agricultural workers who cultivate food in soils. Little is known about how agricultural workers spend their time and how they may contact soil while growing food. Exposure factors are behavioral and environmental variables used in exposure estimation.

**Objectives:**

Our study aimed to derive exposure factors describing how growers engage in different tasks and use those factors to advance the use of time-activity data to estimate soil ingestion exposures among agricultural workers.

**Methods:**

We administered a meso-activity-based, season-specific soil contact activity questionnaire to 38 fruit and vegetable growers. We asked growers to estimate the frequency and duration of six meso-activities and describe how they completed them. We used questionnaire data to derive exposure factors and estimate empirical and simulated exposures to a hypothetical contaminant in soil via incidental ingestion using daily, hourly, and hourly-task-specific ingestion rates.

**Results:**

We generated exposure factors characterizing the frequency and duration of six meso-activities by season, and self-reported soil contact, glove use, and handwashing practices by meso-activity and season. Seasonal average daily doses (ADDs) were similar across all three forms of ingestion rates. No consistent patterns regarding task-specific contributions to seasonal or annual ADDs were observed.

## Introduction

From an exposure standpoint, agricultural work is complex. Agricultural workers who grow food in soils may be exposed to various physical [1, 2], chemical [3–5] and biological hazards [6]. Agricultural labor is a critical input to local, regional, national, and global food systems, but it is not a monolith. Food production yields a variety of specialty and commodity crops and occurs at various scales, using myriad tools, growing methods and practices. Food can be produced for commercial markets at all scales, and for subsistence and by hobbyists. Given the diversity of agricultural contexts, a more nuanced and systematic approach to estimating exposures is needed.

Though related, soil and dust are distinct reservoirs for chemicals in the agricultural context. Several studies have investigated the health impacts of dust exposure [7] in agriculture, but fewer studies have fully characterized the relationship between soil and outdoor dust exposure [8]. A key challenge in this area is determining soil fraction and the environmental conditions driving the cycle between soil and dust for a given exposure scenario. A barrier to fully characterizing this relationship may lie in discord among definitions of soil and indoor and outdoor settled dust provided by the US Environmental Protection Agency (US EPA), interpretations of researchers, and the lived experiences of agricultural workers [9, 10].

Soil is an understudied and underregulated pathway of exposure to chemicals. To date, epidemiologic studies of agricultural workers have focused on inhalation exposures incurred by pesticide applicators without considering soil as a contaminant reservoir with which workers may also have ingestion and dermal exposures [11, 12]. In the US, existing risk assessment guidance for agricultural workers [13, 14] drawing on the Pesticide Handler Exposure Database (PHED) [15] and Agricultural Reentry Task Force (ARTF) [16] for regulating pesticides do not attempt to quantify pesticide exposures resulting from ingestion of or dermal contact with soil. Of all 38 scenarios in the revised PHED (as of May 2021) and 14 scenarios in the ARTF, both databases focus on inhalation and dermal routes of exposure, ignoring the incidental ingestion of soil pathways. Regulatory guidance values for a given contaminant in soil may vary depending on the promulgating agency [17, 18]. To our knowledge, no regulatory agency has developed a recommendation or guideline intended to protect agricultural workers from exposure to soils, though the US EPA Technical Working Group for Lead has put forth recommendations about gardening in lead-contaminated soils [19].

Exposure to chemicals in soil may occur via dermal contact with soil, inhalation of soil particles that become airborne, and incidental ingestion of soil [20]. Of these pathways, the ingestion pathway is the most poorly understood. Existing methods for estimating soil ingestion involve tracer element and biokinetic model comparison methodologies. Activity pattern methodologies use videography to assess micro-activities and activity pattern surveys to assess time engaged in activities relevant to soil exposure [10]. Confidence in existing estimates of soil ingestion rates representing the general US population is low. Little empirical evidence exists to estimate soil ingestion for highly exposed occupational populations like agricultural workers [21, 22]. Because agricultural workers generally spend large amounts of time outdoors and in direct and intentional contact with soil, the use of general population exposure factors may mischaracterize soil exposures incurred by agricultural workers. For example, the US EPA Exposure Factors Program recommends 10 mg/day (central tendency) and 50 mg/day (upper percentile) for estimating soil exposures for adults in the general population but makes no recommendations for highly exposed outdoor worker populations such as agricultural or construction workers [10, 23]. They use a 100 mg/day default soil ingestion rate to derive regional screening levels for contaminants in soils used for adults in residential scenarios and a 330 mg/day default soil ingestion rate for construction workers [24]. It is reasonable to use a higher soil ingestion rate for highly exposed worker populations. Still, no studies have validated these default estimates against real-world exposures, nor have studies verified the appropriateness of these estimates across different occupations—specifically agriculture. Thus, the appropriateness of these general worker soil ingestion estimates for agricultural work is unknown.

Framing agricultural work as a single exposure scenario likely mischaracterizes individuals’ exposure to soil contaminants. Several occupational epidemiology studies have explored matrix approaches to generate estimates of exposures to pesticides in agriculture by task [25–28] and crop type [29]. To our knowledge, this approach has not been applied to characterize exposure to soil contaminants. Given the range of tasks within and the diversity of crops, types of agriculture, and agricultural practices, additional information is needed to understand the range of tasks to assess soil exposures reliably and consistently. A qualitative study of sixteen agricultural workers in Maryland, USA investigated the factors that may impact soil exposure in the agricultural context and yielded a framework describing ten of these environmental, activity, receptor, and timing factors [30]. Despite the advance of this framework in identifying and organizing these key factors, quantitative estimates defining the prevalence, frequency, duration, and magnitude of these factors are needed to advance exposure and risk assessment.

In pursuit of improved characterization of agricultural soil exposure, we describe the design and longitudinal administration of a new soil contact activity questionnaire to collect quantitative estimates of the frequency and duration of key agricultural meso-activities. We summarize and explore these factors, the intensity of soil contact, and the prevalence of behaviors (e.g., personal protective equipment [PPE] use, handwashing) that may impact the extent of soil exposure for each meso-activity. As a case study, we apply the quantitative exposure factors informed by the soil contact activity questionnaire to estimate average daily doses of a hypothetical contaminant in soil via incidental ingestion using daily, hourly, and hourly-task-specific ingestion rates.

## Methods

### Recruitment

We identified fruit and vegetable growers via Maryland’s Best website (https://marylandsbest.maryland.gov) and emailed them invitations to participate in a questionnaire about their farming activities. The Maryland’s Best website is a public database of farm operations maintained by the Maryland Department of Agriculture’s Marketing and Agribusiness Development section to connect producers and consumers. We also contacted growers who previously participated in a qualitative study of soil contact [9] or in the Safe Urban Harvests Study [4, 31]. We contacted 240 growers via email; 5 who responded were ineligible (i.e., not currently growing fruits or vegetables or not located in Maryland); 10 who responded indicated they were not interested; 187 did not respond; and 38 participated in at least one season of data collection. Growers were eligible if they were currently a farm owner/manager, farm employee, or community gardener in Maryland; ≥18 years of age; had completed farm activities related to edible plant production (e.g., planting, harvesting, weeding, mulching) within the past 12 months; and expected to be engaged in farm activities in the upcoming 12 months. We excluded growers who worked on farms that engaged solely in food animal production.

### Data collection

We developed and administered a soil contact activity questionnaire (Supplementary Material) to 38 growers beginning in the spring of 2020. Growers were invited to respond to the same questionnaire each subsequent season (i.e., summer, fall, and winter). Questionnaires were administered between April 2020 and March 2021. Because the questionnaire queries growers about meso-activities conducted in the previous 30 days, administration began 30 days after the start of each season (i.e., administration of the fall questionnaires began on October 22, 2020, 30 days after the first day of fall). Due to restrictions on in-person research due to the COVID-19 pandemic, all questionnaires were administered via telephone. The questionnaire was semi-structured and contained a combination of open- and closed-ended questions. Some growers provided additional information above and beyond their answers; this material was recorded to enhance context. All data were collected and managed using Research Electronic Data Capture (REDCap) tools hosted at the Johns Hopkins Bloomberg School of Public Health [32, 33]. REDCap is a secure, web-based software platform that supports data capture for research studies.

Informed consent was obtained from all growers prior to the administration of the questionnaire. Questionnaire administration times ranged from 5 to 88 min, with a mean of 28 min. Mean questionnaire administration times decreased with each subsequent season (i.e., spring = 40; summer = 27; fall = 28; winter = 18 min). Growers were offered a gift card on an increasing scale (i.e., $5, $10, $15, $20) for each questionnaire completed. All study tools and protocols were reviewed and approved by the Johns Hopkins Institutional Review Board (IRB00009866).

### Soil contact activity questionnaire

The questionnaire was informed by a framework of environmental, activity, timing, and receptor (EAT-R) factors for estimating soil ingestion [30]. Specifically, in this investigation, we aim to generate quantitative and semi-quantitative estimates to characterize further the six meso-activities or tasks (i.e., bed preparation, planting seeds, transplanting, irrigation, weeding, and harvesting) identified by that previous qualitative characterization. These quantitative exposure factors can then be used to inform occupational exposure assessments for agricultural workers. We asked growers to estimate the frequency (days per week) and duration (hours per day) spent engaged in agricultural tasks on-site over a typical month during a given season. We also asked growers to estimate the frequency (days per month) and duration (hours per day) of six specific meso-activities (i.e., bed preparation, planting seeds, transplanting, irrigation, weeding, and harvesting). We also asked growers to estimate what fraction of time their hands were in contact with soil while conducting each task. We then followed up with specific questions about how the task was conducted, i.e., what tools were used, what clothing items were worn, use of PPE, and in what ergonomic position(s) they were in (e.g., kneeling, standing).

We asked all growers, “In the current month, do you recall getting soil in your mouth while working at your farm/garden?” For growers who answered “yes” and reported ingesting soil in the past 30 days, we followed up with two additional questions: 1) “How many days this month do you recall getting soil in your mouth?” and 2) “On a typical day, when you got soil in your mouth, how much soil do you think entered your mouth?” showing them images of varying amounts of soil on spoons (Fig. S1) and asking them to estimate the amount of soil they ingested each day by selecting the image that most closely matched the amount of soil they ingested. For growers who reported ingesting soil in the past 30 days, we showed them images of varying amounts of soil on spoons (Fig. S1) and asked them to estimate the amount of soil/dust they ingested each day by selecting the image that most closely matched the amount of soil/dust they ingested. The amounts of soil on each spoon were based upon EPA’s recommended default exposure factors for daily soil ingestion [10] (10 mg/day = general adult population central tendency; 50 mg/day = general adult population upper percentile; 1000 mg/day = child with soil pica) or dust ingestion (20 mg/day = general adult population central tendency) or soil and dust ingestion (100 mg/day = general adult population upper percentile; 200 mg/day = general child population upper percentile). Two additional options for less than the smallest (<10 mg/day) and greater than the largest amounts (>1000 mg/day) were included. We also queried growers about two events (i.e., incidentally ingesting soil, getting soil on the face) and two behaviors (i.e., sampling produce, and eating a meal or snacks onsite while working) that may increase the likelihood or amount of soil contact. We also asked growers to provide demographic information on themselves (e.g., age, means of compensation) and their farms (e.g., farm size, organic certification status).

### Data analysis

We used Kruskal-Wallis tests to assess differences in growers’ questionnaire responses to four exposure factors: days per month spent at a growing site, hours per month at a site, hours per month engaged in all tasks, across seasons and hours per month engaged in all tasks, across meso-activities. For each exposure factor, we conducted Shapiro-Wilk tests of the distributions to determine whether the distributions were normally or lognormally distributed. We used Pearson correlations to assess correlations between farm size and hours worked per month (both on-site and across all six activities). We used non-parametric Kendall’s tau tests to assess correlations between employment status and hours worked per month (both on-site and across all six activities).

We reviewed all open-ended responses to identify emergent themes, and then through an iterative process SL coded each response according to those themes. Analysis of soil contact activity questionnaire responses was conducted in Excel. Empirical exposure estimation and Monte Carlo simulation, data displays, and hypothesis testing were conducted in Python v. 3.6.

### Exposure assessment

**Fig. 1:**
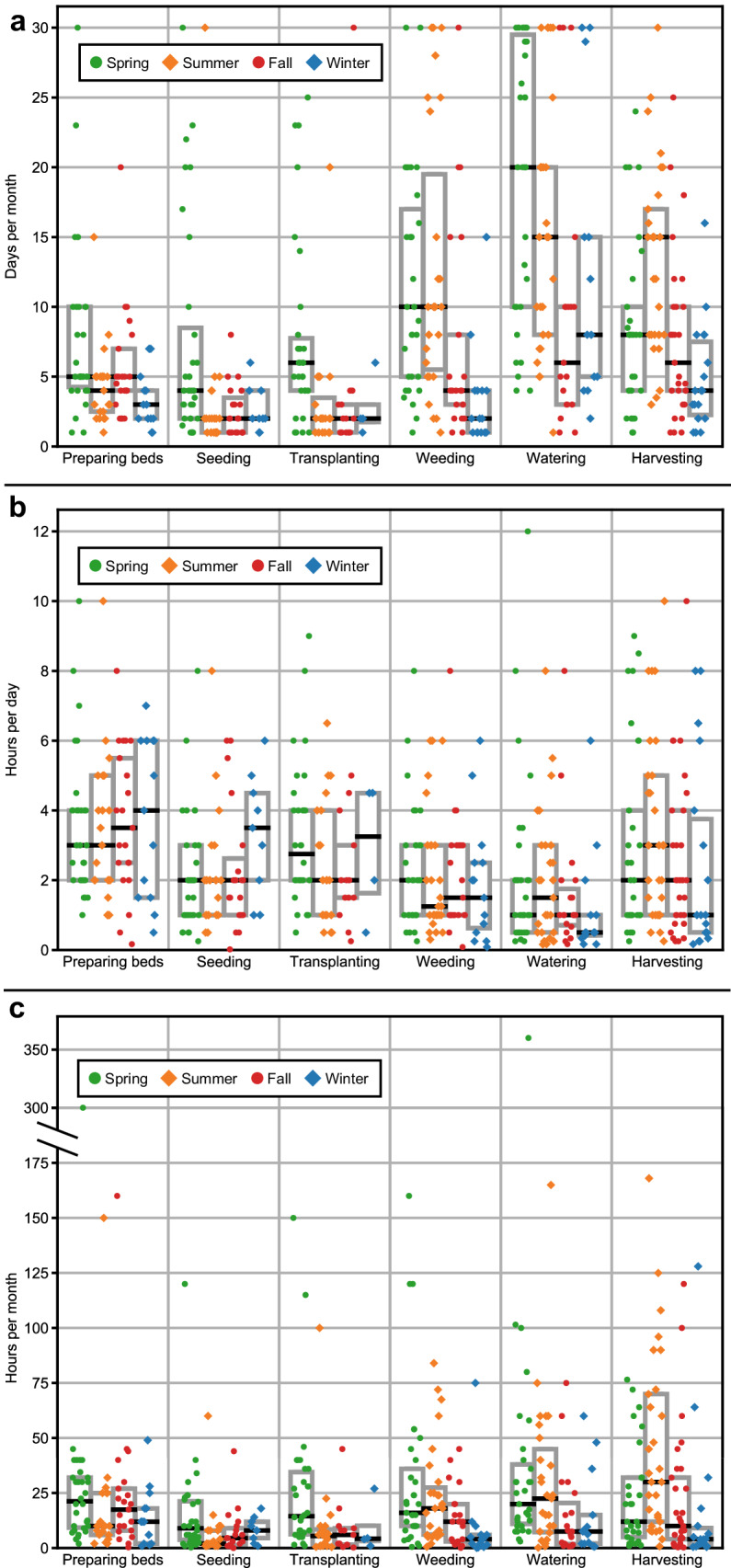
Grower-reported frequency and duration of six meso-activities. **a** Frequency (days per month) of each meso-activity performed, by season. **b** Duration (hours per day) of each meso-activity performed, by season. **c** Cumulative duration (hours per month) of each meso- activity performed, by season. Boxes represent interquartile ranges (Q1-Q3) and black lines represent medians. Results represent only growers who engaged in each activity, i.e., zero values were not factored into box plots. Each dot indicates one grower’s response for a given season.

**Fig. 2:**
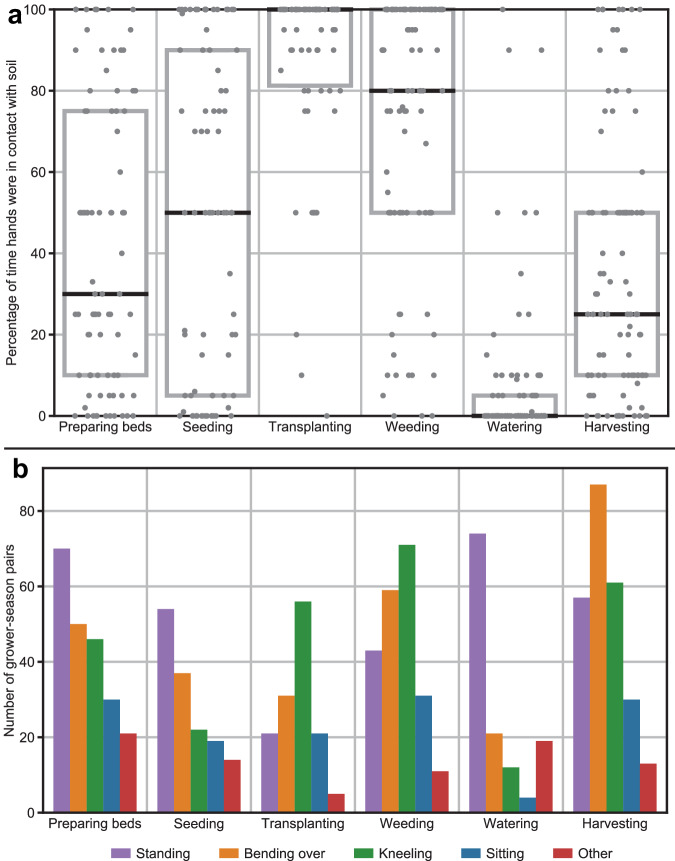
Grower-reported soil contact and ergonomic positioning for six meso-activities. **a** Percentage of time hands were in soil during each activity. **b** Ergonomic positioning during each meso-activity. Responses are not mutually exclusive, i.e., a grower might kneel and sit during the same activity. Examples of “other” positions include: riding on a tractor, squatting, etc. Boxes represent interquartile ranges (Q1-Q3) and black lines represent medians. Each dot represents one grower’s response for a given season. Results represent only growers who engaged in a given activity during a given season.

We estimated exposure by calculating ADDs for each of the 38 growers queried in our study using grower-reported data about the frequency of farming-related meso-activities (Fig. 1), and interactions with soil (Fig. 2). One of the challenges of working with the default soil ingestion rates (e.g., 100 mg/day for the general population and 330 mg/day for highly exposed workers) is the fact that they are presented as daily rates. While daily rates are easier to implement in a regulatory context (i.e., for deriving soil screening levels), they are problematic when used in an occupational context to estimate exposure since they are not easily adaptable for the variable tasks (and their associated soil contact intensities, durations, and frequencies) that constitute agricultural work. For example, the 330 mg/day ingestion rate default is used whether a construction laborer works 8 h or 12 h per day. No adjustments can be made to account for the reasonable assumption that a laborer working more hours would logically incur more exposure than one working fewer than 8 h per day or that a worker may engage in various tasks with different intensities of soil contact. To demonstrate the relevance of incorporating grower-reported frequencies and durations of specific agricultural tasks, we estimated ADDs for each grower who completed questionnaires (empirical) and used Monte Carlo simulation over a 1 month, i.e., (30-day) period using three methods: daily, hourly, and hourly, task-specific soil ingestion rates. The three methods are summarized on GitHub [23].

#### (Traditional) Method 1. Daily soil ingestion

As a baseline, we estimated monthly/seasonal ADDs of a hypothetical chemical using the following equation:

$${ADD}=\frac{{C}_{\frac{{mg}}{{kg}}}* {{IR}}_{\frac{{kg}}{{day}}}* {{EF}}_{\frac{{days}}{{month}}}* {{ED}}_{{months}}}{{{BW}}_{{kgBW}}* {{AT}}_{{days}}}$$

where *C* is the concentration of a hypothetical soil contaminant held constant at 400 mg/kg; *IR* is the daily default soil + dust ingestion rate held constant at 378 mg/day, (adjusted from mg/day to kg/day); and *BW* is the bodyweight of the grower (kgBW). The 378 mg/day soil ingestion rate represents a high-end (95th percentile) estimate derived from a recent study that specifically modeled a high-contact soil scenario [34]. We derived an exposure factor representing the fraction of days worked in a month by multiplying the exposure frequency (EF) or grower-reported days worked per week by 4.35 (i.e., the average number of weeks in a month) to obtain the typical number of days worked per month [23]. The exposure factor was derived using the EF of grower-reported days worked in a typical 30-day period (for that season); an exposure duration (ED) of 1 month, and an averaging time (AT) of 1 month (equivalent to 30.5 days). Bodyweight (BW) was based on the median body weight (kgBW) from the Exposure Factors Handbook [35] associated with the grower’s age bracket and sex [23].

#### Method 2. Hourly soil ingestion

To more precisely account for the variability in the number of hours growers worked per day, we estimated monthly/seasonal ADDs of a hypothetical chemical from outdoor (i.e., working exposures) and indoor (i.e., non-working exposure) using hourly ingestion rates. We derived hourly ingestion rates separately for time spent working outdoors and time spent indoors (not engaged in agricultural work) modeled using ingestion rates in ref. [34]. We derived an hourly ingestion rate for outdoor agricultural work of 45.25 mg/h by dividing the 362 mg/day soil ingestion rate default for growers in a high-contact soil scenario while working outdoors by 8 (assuming the worker works outside for 8 h). Hubbard et al. provide a revised modeled estimate of dust ingestion while indoors of 22 mg/day. To derive an hourly ingestion rate of 1.38 mg/h for dust ingested while indoors (and not engaged in agricultural work), we divided the daily dust ingestion rate (22 mg/day) by 16, assuming that the grower spends all non-working hours indoors.

We estimated seasonal/monthly ADDs for indoor and outdoor exposure to a hypothetical contaminant using the following equation:

$${{ADD}}_{{indoor}\,{or}\,{outdoor}}=\frac{{C}_{\frac{{mg}}{{kg}}}* {{IR}}_{\frac{{kg}}{{hour}}}* {{EF}}_{\frac{{hours}}{{month}}}* {{ED}}_{{months}}}{{{BW}}_{{kgBW}}* {{AT}}_{{hours}}}$$

where *C* is the concentration of a hypothetical soil contaminant (mg/kg), held constant at 400 mg/kg and IR is the hourly default soil ingestion rate held constant at 45.25 mg/hour (adjusted to kg/hour) for hours worked outdoors and 1.38 mg/h (adjusted to kg/hour) for hours not worked and spent indoors. The exposure factor (for outdoor hours) was derived using the EF grower-reported (i.e., number of grower-reported hours worked per day by the number of grower-reported days worked per week, multiplied by 4.35 weeks per month), an ED of 1 month, and an AT of 1 month (equivalent to 730.8 h). The EF (for indoor hours) was assumed to be the complement of outdoor hours and derived by subtracting the grower-reported EF for outdoor hours from 730.8 h. We assumed an ED of 1 month, and an AT of 730.8 h (equivalent to 1 month) for indoor exposures. To convert the average hourly daily dose to an average daily dose we multiplied by 24. Bodyweight was based on the median body weight (kgBW) from the Exposure Factors Handbook [35] associated with the grower’s age bracket and sex. ADDs for both indoor and outdoor exposures were summed.

#### Method 3. Hourly-task-specific soil ingestion

There is variation across agricultural tasks in the rate or intensity of soil contact, but scant research exists to inform precise modifications to hourly ingestion rates by meso-activity. To account for this variation in intensity, we used responses from the grower soil contact activity questionnaires to create scaling factors to adjust the baseline hourly outdoor ingestion rate (45.25 mg/h) to each task, as follows: For each of the six tasks we queried growers about, we asked them to report the percentage of time that involved direct soil contact and we either maintained or adjusted the hourly intake rate up or down accordingly. For example, the mean rate of grower-reported time in contact with soil while engaging in transplanting and weeding was 87% and 72% of the time, respectively [23] so we doubled the baseline hourly soil ingestion rate (45.25 mg/h) for these two tasks to 90.5 mg/h. The mean rate of grower-reported time in contact with soil while engaging in seeding and preparing beds was 49% and 41% of the time, respectively, so we maintained the baseline hourly soil ingestion rate of 45.25 mg/h for these tasks. The mean rate of grower-reported time in contact with soil while engaging in harvesting and watering was 35% and 8% of the time, respectively, so we halved the baseline hourly soil 45.25 mg/h) ingestion rate for these two tasks to (22.63 mg/h). These subjective adjustments to the hourly ingestion rate represent our professional judgment, as directly informed by the grower-reported experiences of soil contact obtained via the soil contact activity questionnaire.

We estimated ADDs of a hypothetical chemical (at 400 mg/kg concentration) over a month using the task-specific hourly ingestion rates and the following equation:

$$\sum {{ADD}}_{{task}\,n}\frac{{C}_{\frac{{mg}}{{kg}}}* {\left({outdoor}\right){IR}}_{\frac{{kg}}{{hour}}}* {{EF}}_{\frac{{hours}}{{month}}}* {{ED}}_{{months}}}{{{BW}}_{{kgBW}}* {{AT}}_{{hours}}}+\frac{{C}_{\frac{{mg}}{{kg}}}* {\left({indoor}\right){IR}}_{\frac{{kg}}{{hour}}}* {{EF}}_{\frac{{hours}}{{month}}}* {{ED}}_{{months}}}{{{BW}}_{{kgBW}}* {{AT}}_{{hours}}}$$

We derived an exposure factor representing the total number of hours worked in a typical month (on each task) by multiplying the number of grower-reported hours worked per day by the number of grower-reported days worked per month (for that task) to obtain the EF. As in method 2, we assumed an ED of 1 month, and an AT of 730.8 h (equivalent to 1 month). To convert the hourly daily dose to an ADD we multiplied by 24. We added the ADDs for each of the 6 tasks to obtain an overall ADD for outdoor exposure for that grower. All hours not accounted for by the grower reported time spent working on each task was assumed indoors and multiplied by the indoor ingestion rate (1.38 mg/h). ADDs for both indoor and outdoor exposures were summed to yield the total daily exposure. As in methods 1 and 2, we used the median body weight (kgBW) from the Exposure Factors Handbook [35] associated with the grower’s age bracket and sex [23].

Complementing the empirical exposure assessments, we simulated exposures among a population of hypothetical growers (*n* = 5000) using Monte Carlo simulation in Python v. 3.6. Simulated exposure assessments follow the three methods described above, adapted as follows:

For all three methods, the bodyweight of each simulated grower was randomly sampled from the distribution provided in the 2017–March 2020 NHANES data [36] filtered to include adults aged 21 and over. To reflect the general United States (US) population, the probability of selecting a given body weight value was weighted by the number of US citizens represented by that datum, as described in Centers for Disease Control and Prevention (CDC) documentation [37].

For the simulated variant of method 1 (traditional, daily soil ingestion), the number of days per month spent at a site, by season, was randomly sampled from the associated values reported in questionnaires. For example, the number of days per month each simulated grower spent at a site during the spring was sampled from the pool of empirical responses specific to the spring. Daily soil ingestion rates were the same as those used in the empirical exposure assessment.

Similarly, for the simulated variant of method 2 (hourly soil ingestion), the number of hours per month spent at a site, by season, was randomly sampled from the associated values reported in questionnaires. Hourly soil ingestion rates were the same as those used in the empirical exposure assessment.

For the simulated variant of method 3 (hourly-task-specific soil ingestion), the number of hours per month engaging in each task, by season, was randomly sampled from the associated values reported in questionnaires. For each task, soil ingestion rates were randomly sampled from a uniform distribution centered around the ingestion rates used in the empirical exposure assessment for method 3 [23]. The ingestion rate used for weeding in the empirical assessment, for example, was twice that of the baseline soil ingestion rate; for the simulated assessment, the ingestion rate for weeding was sampled from a uniform distribution between 1.8 and 2.2 times that of the baseline soil ingestion rate. All task-specific distributions for the simulated assessment similarly ranged from 0.2 times lower to 0.2 times higher than the soil ingestion rates used for the empirical assessment.

## Results

### Grower demographics

Thirty-eight growers completed at least one questionnaire. Twenty-two growers (58%) completed the questionnaire in all 4 seasons; seven (18%) completed the questionnaire in three seasons; six (18%) growers completed the questionnaire in two seasons; three growers completed the questionnaire in one season (8%). Twenty (52%) growers identified with male sex (Table 1). Growers ranged in age from 26–69 with a median age of 40 years. Most growers (71%) were employed more than 35 h weekly. The growers worked on farms ranging in size from 0.4 to 835 hectares. Growers reported a range of job titles, ranging from “Farmer” (3) to “Volunteer” (1) to Project Assistant for Soil Based Research” (1) to “Head Dirt Wearer” (1). Two growers declined to provide a job title. Eleven growers reported a job title combining at least two titles (e.g., Owner/Farmer; Owner/Operator; Owner/Operator/Farm Employee.) Eight (21%) farms were USDA-certified Organic, though 36 (95%) reported using “organic practices.” Farms were in 18 counties (78%) in Maryland. Only one grower from each farm participated.

**Table 1:** Demographics of fruit and vegetable growers at enrollment (*n* = 38) and characteristics of the farms where they work.

Sex	*n* (%)
Male	20 (52)
Female	18 (48)
Smoking status	*n* (%)
Smoker	2 (5)
Non-smoker	36 (95)
Employment status	*n* (%)
Employed, working full-time	27 (71)
Employed, working part-time	1 (3)
Not employed	0 (0)
Retired	4 (11)
Other	6 (16)
Form of compensation	*n* (%)
Salary	2 (5)
Hourly wage	0 (0)
Not paid	10 (26)
Other	26 (68)
Hours worked	*n* (%)
Full time, ≥35 hours/week	28 (74)
Part time, <35 hours/week	8 (21)
Other	2 (5)
Farm size	median (range)
Farm size, in acres	9.5 (0.11–2064)
Area cultivated, in acres	1.8 (0.0005–2056)

### Frequency and duration of meso-activities

Questionnaire responses pertaining to each exposure factor are provided on GitHub [23]. Growers reported spending the greatest number of hours per month on-site in the summer (mean = 204.6; median = 184.9 h per month) and the least hours per month on site in the winter (76.8; median = 54.4 h per month) [23]. All six meso-activities (i.e., bed preparation, planting seeds, transplanting, irrigation, weeding, and harvesting) were reported by at least half of all respondents in the spring and summer. Growers generally spent the greatest number of days per month irrigating (median = 6 days per month in the fall to 20 days per month in the summer), and the greatest number of hours per day preparing beds (medians: ≥3 h), ignoring days when growers did not perform a given meso-activity (Fig. 1) [23]. The meso-activity that occupied the greatest number of hours per month varied by season: preparing beds took the most time in the spring (median = 21 h/month), fall (median = 18 h/month) and winter (median = 12 h/month), but harvesting took the most time in the summer (median = 30 h/month) [23]. Total hours worked per month (for all meso-activities) ranged from 1–360. Medians by season ranged from 5 h per month (winter) to 15 h per month (spring) [23].

### Within-grower reliability of frequency and duration exposure factors

To our knowledge, this is the first collection of time-activity pattern data specific to agricultural workers. To assess the validity of growers’ responses to a series of questions about time spent working and conducting specific tasks, we conducted an analysis to determine the agreement of their responses across several questions in the soil contact activity questionnaire (Fig. S2). First, we asked growers to estimate 1) the frequency and duration of the time spent engaged in any agricultural task (hours per day and days per week) and 2) the frequency and duration of time engaged in six specific tasks (hours per day and days per month). To assess the difference between growers’ responses to these two metrics in each season, we converted both metrics to hours per month and subtracted the total number of hours worked on the six specific tasks from the total number of hours worked onsite. The differences ranged between −226.5 and 330.4 h per month with a mean difference of 62.6 h per month. Only one response was in complete agreement (0 h/month). Eighty-eight (71%) of the responses yielded a positive difference, meaning the estimated time spent on site was greater than the time spent in the six tasks. This suggests that, for most growers, there was some amount of time the growers were on site but not engaging in the six tasks we queried them about. Responses where a greater number of hours engaged in specific tasks than a number of hours spent on site were less prevalent and may indicate concurrent activities (e.g., watering while weeding or weeding while harvesting).

### Meso-activity-specific soil exposure factors

Across all seasons, growers reported transplanting and weeding as the most soil contact-intensive meso-activities—i.e., their hands were in contact with soil on average 87% of the time while transplanting and 72% of the time while weeding (Fig. 2) [23]. One factor that may explain why these meso-activities involved more soil contact is the ergonomic orientation of growers while engaging in each meso-activity. For example, kneeling was the most reported position for transplanting and weeding; standing was the most reported physical orientation for bed preparation, planting seeds and watering; bending over was the most common orientation for harvesting (Fig. 2).

Clothing and use of PPE may also impact the extent of soil contact. Bed preparation and weeding were the two meso-activities in which the greatest percentage of growers (who completed the meso-activity) reported wearing gloves (Fig. S3). However, among growers who wore gloves for a given meso-activity, the mean fraction of time wearing gloves was greater than 65% for all meso-activities. Growers reported wearing gloves for a greater fraction of time while transplanting (mean = 76%) and weeding (mean = 71%), All growers reported wearing long pants and closed-toe shoes for all meso-activities in the fall and winter, though several growers reported wearing sandals for all meso-activities in the spring and summer. Two growers also reported wearing knee pads while conducting meso-activities on their hands and knees.

Over half of all growers reported handwashing after completing all meso-activities (except watering) for all seasons (Fig. S3). Transplanting and harvesting were the two meso-activities in which the greatest percentage of growers reported handwashing immediately after completing the meso-activity.

Reported tool use varied by meso-activity with varying numbers (e.g., 0–>5 tools mentioned) and levels of mechanization. For example, tools for bed preparation ranged from hand tools (e.g., wheel hoes, rakes) to highly mechanized tools (e.g., automatic bed shapers and tractors). Growers described a range of irrigation tools ranging from hand watering tools (e.g., watering cans and hoses) to automatic drip and overhead irrigation systems.

### Non-dietary ingestion events and behaviors

Because incidental ingestion is the pathway hypothesized to contribute the most to soil exposure, we asked growers directly about the occurrence of two related events (i.e., getting soil in the mouth or on the face) and two behaviors (i.e., tasting produce in the field while working and consuming meals or snacks on site). Two growers also reported being current smokers, an activity, unrelated to food production, which may increase the frequency of hand-to-mouth behaviors. These questions were asked generally about their soil contact and were not tied to a specific meso-activity. More growers reported getting soil on their faces in the spring and fall; however, among growers who reported getting soil on their faces, facial contact with soil occurred most frequently in the spring and summer [23]. Among growers who reported ingesting soil in the past 30 days, soil was ingested most frequently during the summer (mean = 9.5 days within the last 30) and spring (mean = 7.9 days within the last 30).

Across all four seasons, sixteen growers estimated ingesting less than 10 mg/day, while 11 growers estimated 10 mg/day and 20 mg/day (Fig. S1). Six growers estimated ingesting 50 mg/day or more, which is the US EPA’s upper percentile default for the general population for soil ingestion for 12 years of age and older [38].

Consuming produce while working in the field was more commonly reported than getting soil on the face or ingesting soil in the last thirty days [23]. Among growers who reported sampling produce while working, it occurred most frequently in the summer, with an average of 15 ingestion events per month, compared to only 6 events per month in the winter. Washing the produce before consuming was reported more often in the winter (100% of the time) than in the spring (mean = 92.5% of the time). Consuming a meal or snacks on site was the least reported potential nondietary ingestion event across all seasons. Sixty percent of participants also reported consuming a meal or snacks onsite each season.

### Differences in and correlations among exposure factors

We used Kruskal-Wallis tests to assess differences in the median values of exposure factors across groups. For days per month at a growing site, hours per month at a site, and hours per month engaged in all activities, we observed statistically significant differences in medians (*p* < 0.05) across seasons. We also observed a statistically significant difference in the median number of hours per month engaging in tasks across the six activities [23].

We used Pearson’s correlation tests to assess correlations among selected continuous variables, and Kendall’s tau to assess correlations between continuous variables and employment status, an ordinal categorical variable where 1 = full time, 2 = part time, and 3 = unemployed, retired, or “other”. Time spent on site, but not time on all six activities, approached a statistically significant (*P* = 0.08) positive correlation with farm size. Employment status was significantly correlated with the number of hours per month at site, whereby full-time growers generally spent more time at the site compared to part-time growers, who in turn spent more time at the site compared to volunteers, retirees, and other unemployed groups [23].

### Additional factors

We asked growers to describe any factors that may cause year-to-year variability in their responses. Growers most described weather as a reason for variation in the conduct of specific meso-activities or the nature of their soil contact from the current year compared to the previous year. Workforce differences (e.g., additional staff or fewer onsite volunteers) and changes in production practices (e.g., not planting cover crops) were also frequently mentioned. As data collection occurred during the 2020 growing season, during the COVID-19 pandemic, growers mentioned the COVID-19 pandemic as a specific driver of year-to-year variability. Notably, the pandemic impacted other factors such as increased hygiene practices and consumer demand and decreasing the size of the on-site workforce and difficulty obtaining PPE due to high demand within the healthcare industry.

### Application: Estimated exposures using task-specific exposure factors from soil contact activity questionnaire

**Fig. 3:**
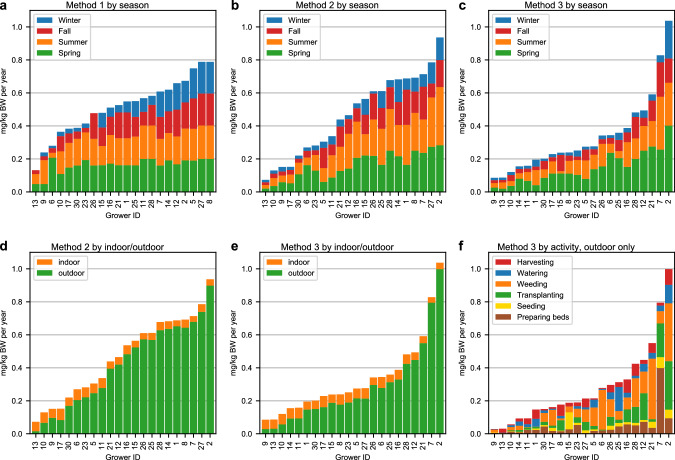
Total annual average daily doses (ADDs) estimated using three methods for estimating soil ingestion (*n* = 22) Each stacked bar chart shows the total annual exposure for a single grower who completed a soil contact activity questionnaire in all four seasons. **a**–**c** show the annual exposure by season across three methods (1 = daily ingestion rate; 2 = hourly ingestion rate; 3 = hourly task-specific ingestion rate). **d**, **e** show the total and relative estimated exposure originating from hours spent indoors vs. outdoors for methods 2 and 3. **f** shows the total and relative exposure originating from each of six meso-activities using method 3.

**Fig. 4:**
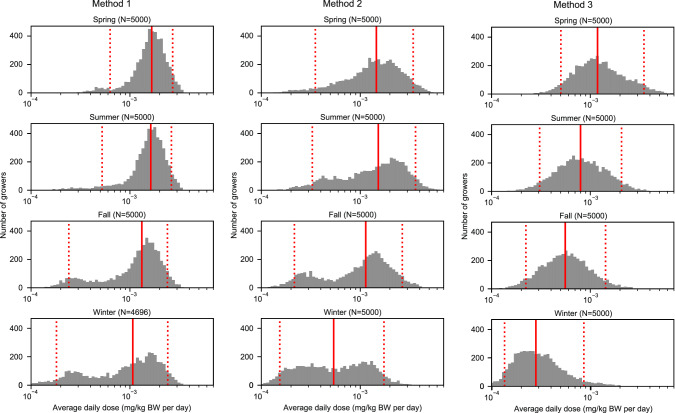
Seasonal average daily doses (ADDs) by soil ingestion method among simulated growers (*n* = 5000). Each histogram shows the distribution of seasonal ADDs for 5000 simulated growers using one of three types of soil ingestion rates. The left column shows seasonal ADDs estimated using daily soil ingestion rates (method 1). The center column shows seasonal ADDs estimated using hourly soil ingestion rates (method 2). The right column shows seasonal average daily doses estimated using hourly-task-specific soil ingestion rates (method 3). The solid red line represents the median and the dotted red lines represent the 5th and 95th percentiles.

We estimated total annual exposures for all growers who completed questionnaires in all four seasons using all three methods (daily, hourly, and hourly-task-specific ingestion rates) (Fig. 3; Figs. S4–5) [23]. All of these exposure estimates represent exposure to a hypothetical soil contaminant assumed at constant of 400 mg/kg. Cumulative annual exposures were generally greatest in method 1 (traditional, daily soil ingestion rates) and lowest in method 3 (hourly-task-specific soil ingestion rates). Across all three methods, most of the annual exposure occurred in the spring and summer seasons (Fig. 4) [23]. Under method 3, there were no discernable patterns of which activities contributed the most (or least) to annual exposure. The distribution of ADDs for preparing beds and seeding in the winter were normally distributed.

We estimated seasonal and task specific ADDs for a hypothetical population of growers using a Monte Carlo sampling approach and data from all growers who completed the soil contact activity questionnaire using all three methods (daily, hourly, and hourly-task-specific ingestion rates). Across all three methods, the mean ADDs were lowest in the winter and fall and highest in the summer and spring. Only in method 2 was the mean ADD greatest in the summer (not the spring.) All methods yielded comparable distributions with means and medians of seasonal ADDs of similar orders of magnitude, though the underlying shapes of the distributions are worth noting. For example, method 1 yielded distributions that were skewed left for all seasons, whereas method 2 yielded seasonal distributions that were less skewed and covered larger ranges than method 1. No ADDs were normally or lognormally distributed for any season under any of the three methods.

**Fig. 5:**
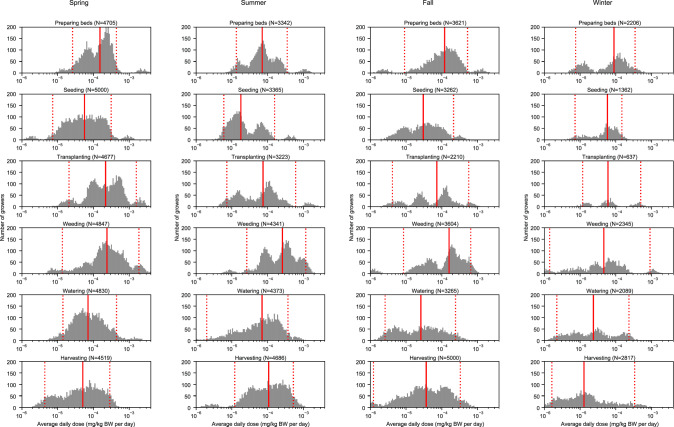
Seasonal average daily doses (ADDs) by meso-activity and season among simulated growers (*n* = 5000) using method 3 (hourly-task-specific soil ingestion rates). Each histogram shows the distribution of seasonal, task-specific ADDs for 5000 simulated growers using hourly-task-specific soil ingestion rates (method 3). The solid red line represents the median and the dotted red lines represent the 5th and 95th percentiles.

Within method 3, we explored the ADDs attributable to each season-task combination (Fig. 5) [23]. The means of seasonal and task-specific ADDs were more variable than the overall seasonal ADDs, ranging 2 orders of magnitude (i.e., 1.72 × 10^−5 ^mg/kgBW/day for transplanting in the winter to 4.32 × 10^−4^ mg/kgBW/day for weeding in the spring.) Season-task ADD distributions yielded several distinct shapes. The distribution of transplanting ADDs were multi-modal in all seasons [23].

## Discussion

We generated season and meso-activity-specific exposure factors that describe the extent of soil exposure among fruit and vegetable growers in Maryland and are a first step towards a more concerted effort to derive public and occupational health guidelines for soil contaminants in the agricultural sector. Our findings demonstrate notable variation in both the frequency and duration of meso-activities and growers’ perceived intensity of soil exposure within and across meso-activities. We also demonstrate the utility of this novel, quantitative task-specific exposure factors (i.e., frequency and duration) by modifying traditional soil ingestion exposure models to estimate seasonal and task-specific average daily doses of a hypothetical contaminant in soil.

### Soil contact activity questionnaire and task-specific exposure factors

In our sample of growers, we found that irrigation is the meso-activity that occupies the greatest amount of time each season. Growers reported that irrigation was conducted on the greatest number of days each month (i.e., median frequency = 20 days in the spring; 10 days in the summer), but bed preparation was conducted for a longer period of time (median duration = 3 h per day in the spring; 2.5 h per day in the fall) on each day it is completed. Our data also provide information on growers’ perceived intensity of soil contact within and across meso-activities and the prevalence of soil exposure reduction behaviors which could be used to guide interventions to reduce soil contaminant exposures on farms. Transplanting and weeding were the meso-activities with the greatest reported soil contact. Given our time-activity findings, exposure scientists may recommend interventions to reduce exposure focused on irrigation, as that was the meso-activity in which the greatest number of hours were spent. Yet, others may recommend interventions targeting weeding and transplanting, as those were the meso-activities that involved the greatest reported soil contact for growers, despite being done less frequently and over a shorter duration. Our findings suggest that neither approach alone may be appropriate in practice. We also identified and defined meso-activities according to grower-reported definitions of each meso-activity. For some activities, the specific actions involved in the task may vary substantially within and across growers. Irrigation, for example, may include actions on a given day ranging from installing and maintaining drip irrigation systems, hand watering with a can or hose, or turning on a pre-installed system. Thus, our findings emphasize the need for a complete and more nuanced investigation of meso-activities within agriculture, which may improve estimations of soil ingestion for risk assessment and inform interventions to reduce soil exposure.

We also observed variability in growers’ self-reported behaviors which may modulate the intensity of soil exposure within a given meso-activity. Given our methods, these findings characterize soil ingestion that occurs both when growers were aware and report getting soil in their mouths and when growers take a deliberate action but unintentionally ingest soil (e.g., growers reporting sampling produce without washing it prior to consumption). With this method, we cannot differentiate or characterize soil ingestion that is intentional (e.g., pica behavior, or intentional consumption of soil) or that which is subconscious (i.e., that growers are not directly aware of). Both of these types of ingestion may also contribute significantly to exposure.

### Hourly-task-specific exposure assessment

Given the variability in the grower-reported time engaged in six different agricultural tasks, we build upon a rigorous modeling study using the US EPA Stochastic Human Exposure Dose- Simulation models to estimate ingestion rates for high soil exposure scenarios [34] to pioneer a more nuanced framework for estimating of soil ingestion involving a more detailed use of time-activity pattern data (both task-specific and non-working time). Our three methods for estimating seasonal and seasonal task-specific ADDs (both empirically and simulated) yielded similar estimates, i.e., generally of the same order of magnitude, with method 3 generally yielding the lowest annual ADDs. These results are expected, given the EF used in method 2 (hours per month worked on site) for most growers queried were more than the EF used in method 3 (hours per month engaged in all 6 meso-activities.) Thus, in method 3, any hours not spent engaged directly in one of the six meso-activities queried in this study were assumed to be spent indoors and not engaged in any agricultural work and assigned an hourly soil intake rate much lower than would be typical for outdoor exposures. This assumption has likely underestimated exposure for growers who were engaged in meso-activities beyond one of the six investigated (e.g., animal care, or construction).

Future investigations should consider ways to improve survey design to query growers about other tasks beyond the six included in this study and characterize the prevalence of concurrent tasks, both of which may impact the accuracy of exposure estimates.

We used a combination of grower-reported task-specific time-activity pattern data and soil contact intensity to inform the translation of a default daily ingestion rate into hourly and hourly-task-specific ingestion rates using our professional judgment. Confidence in the default soil ingestion rates for the general population, and highly exposed agricultural workers remains low. Further research to quantify and validate the daily, hourly and hourly-task-specific incidental ingestion rates we used in this assessment is warranted.

### Strengths and limitations

A key strength of our method was the open-ended collection of information within the soil contact activity questionnaire, which increased our ability to collect novel information about growers’ meso-activities and growing methods. We suspect that our estimates of growers’ conduct of irrigation may be inconsistent for time in contact with soil, given that many growers described multi-tasking (e.g., turning on an irrigation system and then switching to another meso-activity while the system runs), or conducting additional tasks simultaneously while irrigating (e.g., weeding while irrigating). If growers conducted more soil-intensive tasks while watering, we underestimate exposure, but if growers conducted a less soil-contact-intensive task, we overestimate exposure. Both scenarios are plausible and were described by growers. In addition, we also learned that when asking about handwashing practices, the next meso-activity may be a stronger indicator of whether a grower would wash their hands than the current meso-activity; several growers explained scenarios in which they would wash their hands after preparing beds if they were eating lunch or harvesting next, but not if they were moving directly to a more soil-contact-intensive meso-activity (such as transplanting).

Anecdotally, our select open-ended questions allowed for spontaneous participant feedback in real time about perceived ambiguity and their understanding of the standardized questions. For example, when asking growers to estimate the amount of soil they got in their mouths on a typical day, some growers responded “no” because they considered the grittiness in their teeth “dust” rather than soil.” This observation highlights the variability in growers’ notions of the environmental media relative to exposure scientists’ definitions and the importance of establishing a shared vocabulary when assessing both soil and dust exposures in this context.

To balance rigorous data collection with growers’ ability to recall (in detail) the frequency and duration of specific meso-activities as well as the context and means of completing them, we designed the questionnaire to query growers about their meso-activities over only the previous thirty days. Due to the staggered nature in which we administered the questionnaire over four seasons, we are unable to determine whether the observed within-season variability is due to grower differences or attributable to weather or other timing-related factors. In addition, because we prioritized data collection regarding growers’ overall time and behavioral tasks and did not ask growers to conduct time-activity diaries, we are uncertain about the order and specific timing of meso-activities on a given day and how these may impact soil contact.

We observed considerable variability in time spent engaging in specific meso-activities, and the means for doing so. Some of this variability may be driven by the size of the farm operation, or the number of hours worked (e.g., full or part-time), specific plants cultivated, tools used, or specific regional differences in climate or growing practices. Most growers queried were the farm manager or owner/operator, and each grower queried worked at a unique farm or garden. Thus, our findings cannot inform discussions of how soil contact, hours worked, or behavioral factors may vary across several growers at the same time (e.g., season) and place (e.g., farm). This may be of particular interest at larger farms where tasks may be distributed differently among several workers at a site, resulting in differential exposure across workers. Additional research is needed to discern the extent to which potential differences in soil exposure across workers is driven by environmental (e.g., farm context), activity, timing or other behavioral factors specific to each receptor [30].

We use the term “growers” throughout this manuscript to maintain emphasis on the meso-activities pertaining to the direct cultivation of plants. This emphasis may limit the relevance of our findings to broader notions of agricultural work. Our findings may be relevant to other small-scale fruit and vegetable growers in other states, though additional research is needed to assess the generalizability of our findings to large-scale commodity growers as there may be notable differences in meso-activities in these operations. Specifically, we anticipate that the use of mechanized equipment may differentially modify exposure. We also included very small-scale growers, many of them self-identifying as “community gardeners,” in our study because they fit our eligibility criteria in response to the increasing popularity of urban agriculture and community gardening. While hobbyist community gardeners and full-time migrant farmworkers may have different exposure experiences, we hope others will consider both groups, and the full range of growers who grow our food and interact with soils.

Our data provide helpful proxies for calibrating soil exposure estimates but rely exclusively on growers’ recall and reporting of meso-activities and conscious behaviors across tasks. We asked growers to estimate how much soil they think they ingested on a typical day but could not validate these estimates with direct measurements. A previous investigation of soil loading found adult volunteers generally reported 10 mg of soil in the mouth was notably unpleasant and easily detectable in a single event providing a reasonable benchmark for perceptible amounts of soil in the mouth [39]. We hope additional research will be conducted to validate growers’ self-reported activities and behaviors including self-reported incidental ingestion and other meso-activities beyond the six we investigated that may occupy a significant amount of the growers’ hours working on site.

### Future research directions

Future studies could use these findings on growers’ self-reported soil contact within meso-activities to prioritize which meso-activities may be most appropriate for direct observation for quantifying subconscious soil exposure-relevant micro-activities. Coupling data on the frequencies of hand -to mouth-and face-to-mouth micro-activities with existing studies of soil loading and transfer rates across meso-activities would radically advance soil exposure modeling[21, 22, 39]. For example, while glove use may initially reduce dermal exposure to soil on the hands, some growers may remove soiled gloves with their teeth, or wipe a sweaty brow more with a gloved hand more frequently than an ungloved hand, resulting in greater rates of incidental soil ingestion than would occur if gloves were not worn. Better understanding and quantification of such micro-activity pathways is needed to account for the full extent of soil exposure. Information on such micro-activities coupled with information generated from time-activity diaries (administered on a larger scale) will improve estimates of soil contact and ingestion. Advances in research identifying novel tracers may also help validate estimates of soil and dust ingestion in the agricultural sector and beyond [40, 41].

Though our exposure assessments did not yield large differences in ADDs using three types of soil ingestion rates (daily, hourly and hourly-task-specific), we believe the underlying methods, accompanied by an improved understanding of task-specific variations in soil ingestion rates constitute a critical advance towards a more rigorous and precise characterization of soil exposure in the agricultural context. Traditional soil exposure models include a single daily ingestion rate for a given scenario (method 1). We pioneer a data-driven approach, rooted in a time-activity pattern methodology that differentiates workers’ exposures incurred while on site and off-site, (method 2) and attributable to the amount of time spent engaged in specific meso-activities (method 3). Future studies should characterize the frequency and duration of distinct agricultural meso-activities. These models do not currently account for the intensity, positioning, and orientation of growers in relation to soil that may impact soil exposure within and across each of the meso-activities investigated. Specifically, data are unavailable to inform variations in the soil ingestion rate; different meso-activities likely correspond to different ingestion rates. In the absence of these data, we used the percentage of time growers in this study reported direct hand contact with soil for each task to inform adjustments to hourly-task-specific ingestion rates. Additional research to understand this variation would improve the rigor of such exposure estimates. Also, our modeling does not consider behaviors (e.g., the frequency, duration, and timing of handwashing) that may reduce soil exposures among growers.

Traditionally, pesticide risk assessment and regulation have focused on exposures incurred during the direct application of pesticides via inhalation and dermal pathways [42] without explicit consideration of soil exposure [43]. Given the persistence of pesticides in soils after application [11], our approach to estimating soil exposure provides the framework and tools needed to develop a more comprehensive assessment of pesticide exposure. For example, though not quantitatively incorporated in the models demonstrated here, consideration of ergonomic positioning while engaging in specific meso-activities provides more information about the relative location of a worker’s breathing zone and may improve estimates of inhalation exposures to pesticides incurred during and after application. Including additional pathways of pesticide exposure via ingestion of soil in risk assessments of pesticides facilitates a more rigorous estimation of chronic exposures and consideration of non-applicator agricultural worker exposures in pesticide risk assessment.

## Conclusion

We generated estimates of a variety of activity, timing, and receptor factors impacting soil exposure among agricultural workers and used them to model exposures via incidental ingestion. Our method characterized numerous conscious activities and behaviors that growers engage in, though future research efforts should advance methods that characterize meso-activity-specific ingestion rates and the subconscious factors that may also contribute to soil exposure. Our findings estimate hypothetical soil exposures using these novel exposure factors and demonstrate how meso-activity data could support more robust estimates of soil ingestion to support further development of regulatory guidance values for soil contaminants specific to agricultural workers.

## Supplementary information

Supplemental Material

## Data Availability

De-identified questionnaire data, summary distributions of exposure factor data collected via the soil contact activity questionnaire, and summary distributions of the Monte Carlo exposure assessment are available on GitHub [23].
