# Supplementary material for: Application and demonstration of meso-activity exposure factors to advance estimates of incidental soil ingestion among agricultural workers
Source: J Expo Sci Environ Epidemiol. 2024 May 17;35(2):303–14. doi: 10.1038/s41370-024-00671-0 (PMC12009730; doi:10.1038/s41370-024-00671-0)
Supplement: Supplementary file 1 — Supplemental Material [file 41370_2024_671_MOESM1_ESM.docx]

**Supplemental Material**

**Novel meso-activity exposure factors to advance estimates of incidental soil ingestion among agricultural workers**

Sara N. Lupolt, Brent F. Kim, Jacqueline Agnew, Gurumurthy Ramachandran, Thomas A Burke, Ryan David Kennedy, Keeve E. Nachman

**Table of Contents**

**Supplemental information.**

Soil contact activity questionnaire

**Supplemental figures**

**Figure S1.** Assessment of grower-reported soil ingestion rates

**Figure S2.** Differences between grower-reported time spent on site and total time spent engaged in six meso-activities (hours per month) for four seasons

**Figure S3.** Frequency of grower-reported exposure reduction behaviors (glove-wearing and handwashing) during/after six meso-activities

**Figure S4.** Seasonal average daily doses by soil ingestion method among growers who responded to the soil contact activity questionnaire

**Figure S5.** Seasonal average daily doses by meso-activity among growers who completed the soil contact activity questionnaire using hourly, task-specific soil ingestion rates

### Soil Contact Activity Questionnaire

**Demographics**

1. What is your sex?

Male

Female

1. In what year were you born?
2. Which of the following categories best describes your employment status?

Employed, working full-time

Employed, working part-time

Not employed, looking for employment

Not employed, NOT looking for employment

Retired

Other (please specify)

1. Which of the following categories best describes how you are compensated for work at your farm/garden?

Salary

Hourly wage

Not paid

Other (please specify)

1. Which of the following categories best describes how often you work at your farm/garden?

Full time, ≥35 hours/week

Part time <35 hours/week

Other (please specify)

1. What farms/gardens are you affiliated with?
2. What is your job title?
3. How large is your primary farm/garden (in acres)?
4. How large is your growing area on your primary farm/garden(in acres)?
5. Is your primary farm/garden USDA certified organic?

Yes No

1. Do you generally use practices that are considered USDA certified organic?

Yes No

1. Do you smoke cigarettes or e-cigarettes while working at your farm/garden?

Yes No

**Overall activities**

1. During the current season [insert three months of interest], how many **days per week** are you typically present at your farm/garden?

______ (number)

1. For each day you are on site this season [insert three months of interest], how many **hours per day** are you typically present at your farm/garden?

______ (number)

1. During the current season [insert three months of interest], how many **hours per week** are you present at your farm/garden?
   1. How many of these hours are you directly engaged in food production tasks?

______ (number)

- 1. How many of these hours are you NOT directly engaged in food production tasks?

______ (number)

**Soil ingestion events**

1. In the current month, do you recall getting soil in your mouth while working at your farm/garden?

Yes No

- 1. How **many days this month** do you recall getting soil in your mouth?

*(for administration via phone) For the next question, I am going to send you an image via text/email. What is your preference?*

*(for administration via phone) Please confirm your phone/email, and let me know when you have received the image.*

- 1. On a typical day, when you got soil in your mouth, how much soil do you think entered your mouth? Please refer to the image presented.

Less than A

A

B

C

D

E

F

More than F

- 1. Please describe what you are typically doing when you get soil in your mouth? (short answer, open ended)

1. In the current month, do you recall getting soil on your face?

Yes No

- 1. If yes, how many times this month you recall getting soil on your face?
  2. Please describe what you are typically doing when you get soil on your face? (short answer, open ended)

1. In the current month, did you ever consume produce items (grown onsite) while at your farm/garden?

Yes No

- 1. If yes, how many times in the current month have you consumed produce items grown on site?
  2. Do you ever wash the produce before consumption?

Yes No

If yes, how often do you typically wash the produce before consumption? (use sliding scale)

0% never

100% - all the time

1. In the current month, did you ever consume a meal or snacks (brought in from offsite) while at your farm/garden?

Yes No

- 1. If yes, how many times in the current month have you consumed a meal or snacks on site?

**Specific activities**

1. In the current month, did you (or do you plan to) do *any* ***bed preparation or maintenance*** at your farm/garden?

Yes No

- 1. If yes, **how many days this month** do you do typically do *bed preparation or maintenance*?

______________ (integer)

- 1. On the days that you do *bed preparation or maintenance***, how many hours** does it typically take per day?

_____________(number)

- 1. When you do *bed preparation or maintenance*, are you typically (select all that apply)

Standing

Bending over

Kneeling

Sitting on the ground

Other _________________

- 1. When you do *bed preparation or maintenance*, how much of the time are your hands typically in contact with soil? (use sliding scale)

0% no soil contact

25% some of the time

50% half of the time

75% most of the time

100% constant contact

- 1. Please describe any tools you typically use to assist you with bed preparation or maintenance? (open ended, short answer response)
  2. Do you ever wear gloves when *you do bed preparation or maintenance*?

Yes No

If yes, how much of the time do you typically wear gloves? (use sliding scale)

0% never

100% - all the time

- 1. When you do *bed preparation or maintenance* select the clothing items you typically wear (select all that apply)
  2. Do you typically wash your hands immediately after *you do bed preparation or maintenance*?

Yes No

If yes, how much of the time do you typically wash your hands immediately after bed preparation or maintenance? (use sliding scale)

0% never

100% - all the time

1. In the current month, did you (or do you plan to) do ***planting*** at your farm/garden?

Yes No

- 1. If yes, did you plant seeds?

Yes No

- - 1. If yes, please list the items planted (short answer, open ended).
    2. In the current month, how many **days** did you plant seeds?

_______ (integer)

- - 1. On the days that you planted seeds, how many **hours per day** were you typically planting seeds?

_____________(number)

- - 1. When you are *planting seeds*, are you typically (select all that apply):

Standing

Bending over

Kneeling

Sitting on the ground

Other _________________

- - 1. When you are planting seeds, how much of the time are your hands typically in contact with soil? (use sliding scale)

0% no soil contact

25% some of the time

50% half of the time

75% most of the time

100% constant contact

- - 1. Please describe any tools you typically use to assist you with planting seeds? (open ended, short answer response)
    2. Do you ever wear gloves when *planting seeds*?

Yes No

If yes, how much of the time do you typically wear gloves when planting seeds? (use sliding scale)

0% never

100% - all the time

- - 1. This month, when you plant seeds, select the clothing items you typically wear (select all that apply):

Long pants Shorts

Long sleeves Short sleeves

Close toed shoes Sandals

Hat

Other

- - 1. Do you typically wash your hands immediately after *planting seeds*?

Yes No

If yes, how much of the time do you typically wash your hands immediately after planting seeds? (use sliding scale)

0% never

100% - all the time

- 1. If yes, did you plant transplants?
     1. If yes, please list the items transplanted:
     2. In the current month, **how many days this month** did you transplant?

_______ (integer)

- - 1. On the days you transplanted plants, how many **hours per day** typically did you transplant?

_____________(number)

- - 1. When you are *transplanting*, are you typically (select all that apply):

Standing

Bending over

Kneeling

Sitting on the ground

Other _________________

- - 1. When you are planting transplant, how much of the time are your hands typically in contact with soil? (use sliding scale)

0% no soil contact

25% some of the time

50% half of the time

75% most of the time

100% constant contact

- - 1. Please describe any tools you typically use to assist you with transplanting? (open ended, short answer response)
    2. Do you ever wear gloves when *transplanting*?

Yes No

If yes, how much of the time do you typically wear gloves? (use sliding scale)

0% never

100% - all the time

- - 1. When you transplant, select the clothing items you typically wear:

Long pants Shorts

Long sleeves Short sleeves

Close toed shoes Sandals

Hat

Other

- - 1. Do you typically wash your hands immediately after *transplanting*?

Yes No

If yes, how much of the time do you typically wash your hands immediately after transplanting? (use sliding scale)

0% never

100% - all the time

1. In the current month, did you (or do you plan to) do *watering* at your farm/garden?

Yes No

- 1. If yes, how many days this month do you do *watering*?

______________ (integer)

- 1. On the days you do water, many hours per day were you typically watering?

_____________(number)

- 1. When you do *watering*, are you typically (select all that apply)

Standing

Bending over

Kneeling

Sitting on the ground

Other

- 1. When you do *watering*, how much of the time are your hands in contact with soil?

(use sliding scale)

0% no soil contact

25% some of the time

50% half of the time

75% most of the time

100% constant contact

- 1. Please describe any tools you use to assist you with watering? (open ended, short answer response)
  2. Do you ever wear gloves when *watering*?

Yes No

If yes, how much of the time do you typically wear gloves? (use sliding scale)

0% never

100% - all the time

- 1. When you do *watering* select the clothing items you are most likely to wear:

Long pants Shorts

Long sleeves Short sleeves

Close toed shoes Sandals

Hat

Other

- 1. Do you typically wash your hands immediately after *watering*?

Yes No

If yes, how much of the time do you typically wash your hands immediately after watering? (use sliding scale)

0% never

100% - all the time

1. In the current month, did you (or do you plan to) do *weeding* at your farm/garden?

Yes No

- 1. If yes, how many days this month do you do *weeding*?

______________ (integer)

- 1. On the days you do weed, how many hours per day are you typically weeding?

_____________(number)

- 1. When you do *weeding*, are you typically (select all that apply)

Standing

Bending over

Kneeling

Sitting on the ground

Other

- 1. When you do *weeding*, how much of the time are your hands in contact with soil?

(use sliding scale)

0% no soil contact

25% some of the time

50% half of the time

75% most of the time

100% constant contact

- 1. Please describe any tools you use to assist you with weeding? (open ended, short answer response)
  2. Do you ever wear gloves when *weeding*?

Yes No

If yes, how much of the time do you typically wear gloves? (use sliding scale)

0% never

100% - all the time

- 1. When you do *weeding* select the clothing items you typically wear (select all that apply):

Long pants Shorts

Long sleeves Short sleeves

Close toed shoes Sandals

Hat

Other

- 1. Do wash your hands immediately after *weeding*?

Yes No

If yes, how much of the time do you typically wash your hands immediately after weeding? (use sliding scale)

0% never

100% - all the time

5. In the current month, did you (or do you plan to) do *harvesting* at your farm/garden?

Yes No

- 1. If yes, list items harvested: (open ended, short answer.)
  2. How many days this month do you do *harvesting*?

______________ (integer)

- 1. On the days you do harvest, how many hours per day are you typically harvesting?

_____________(number)

- 1. When you do *harvesting*, are you typically (select all that apply)

Standing

Bending over

Kneeling

Sitting on the ground

Other

- 1. When you do *harvesting*, how much of the time are your hands in contact with soil?

(use sliding scale)

0% no soil contact

25% some of the time

50% half of the time

75% most of the time

100% constant contact

- 1. Please describe any tools you use to assist you with harvesting? (open ended, short answer response)
  2. Do you ever wear gloves when *harvesting*?

Yes No

If yes, how much of the time do you typically wear gloves? (use sliding scale)

0% never

100% - all the time

- 1. When you do *harvesting* select the clothing items you are most likely to wear:

Long pants Shorts

Long sleeves Short sleeves

Close toed shoes Sandals

Hat

Other

- 1. Do you typically wash your hands immediately after *harvesting*?

Yes No

If yes, how much of the time do you typically wash your hands immediately after harvesting? (use sliding scale)

0% never

100% - all the time

6. In the past month, did you (or do you plan to) do any other tasks at your farm/garden? (Asked up to 3 times).

Yes No

a. If yes, please describe an additional task? (open ended short answer response)

- 1. How many times this month did you do this task?

______________ (integer)

- 1. On days you do this task, how many hours per day does it typically take?

_____________(number)

- 1. When you do *this task*, are you typically (select all that apply)

Standing

Bending over

Kneeling

Sitting on the ground

Other _________________

- 1. Please describe any tools you typically use to assist you with this task? (open ended, short answer response)

f. When you do *this*, how much of the time are your hands in contact with soil?

(use sliding scale)

0% no soil contact

25% some of the time

50% half of the time

75% most of the time

100% constant contact

- 1. Do you ever wear gloves when you do this task?

Yes No

If yes, how much of the time do you typically wear gloves? (use sliding scale)

0% never

100% - all the time

- 1. When you do *this* task, select the clothing items you are most likely to wear:

Long pants Shorts

Long sleeves Short sleeves

Close toed shoes Sandals

Hat

Other

- 1. Do you typically wash your hands immediately after *this task*?

Yes No

If yes, how much of the time do you typically wash your hands immediately after this task? (use sliding scale)

0% never

100% - all the time

1. Please describe any factors or conditions in the past month that have made your answers to any of the questions different than what you would have answered a year ago. (short answer, open ended.)

**Figure S1.** Assessment of grower-reported soil ingestion rates


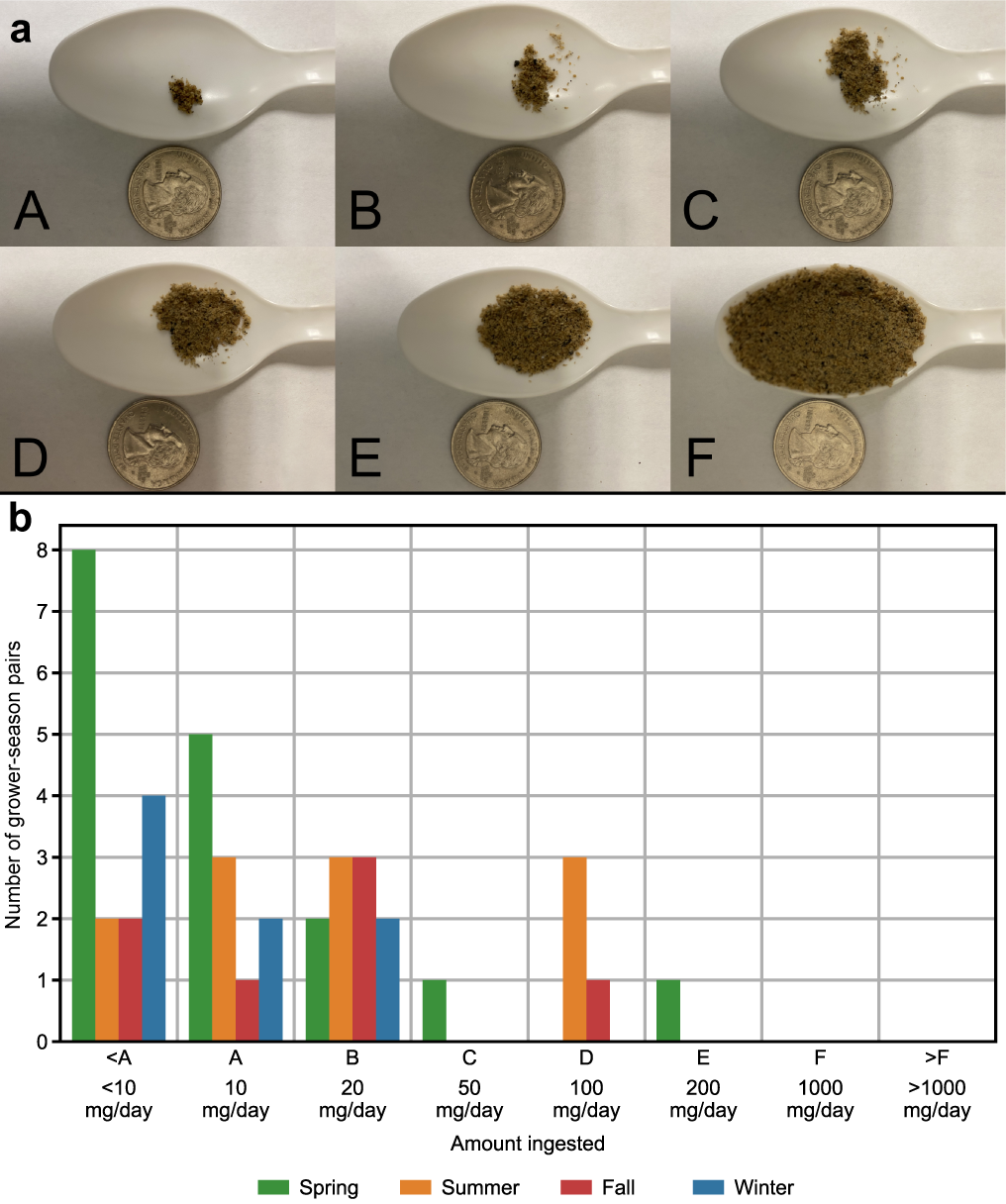


(a) Images of amounts of soil presented to growers who reported ingesting soil in the past thirty days. The amounts are recommended estimates of ingestion rates for use in risk assessments from the US EPA Exposure program ranging from less than 10 mg/day to more than 1,000 mg/day. (b) Number of growers who reported ingesting each amount, by season. Results represent only those growers who also reported ingesting soil in the past 30 days (from date of questionnaire completion).

**Figure S2.** Differences between grower-reported time spent on site and total time spent engaged in six meso-activities (hours per month) for four seasons


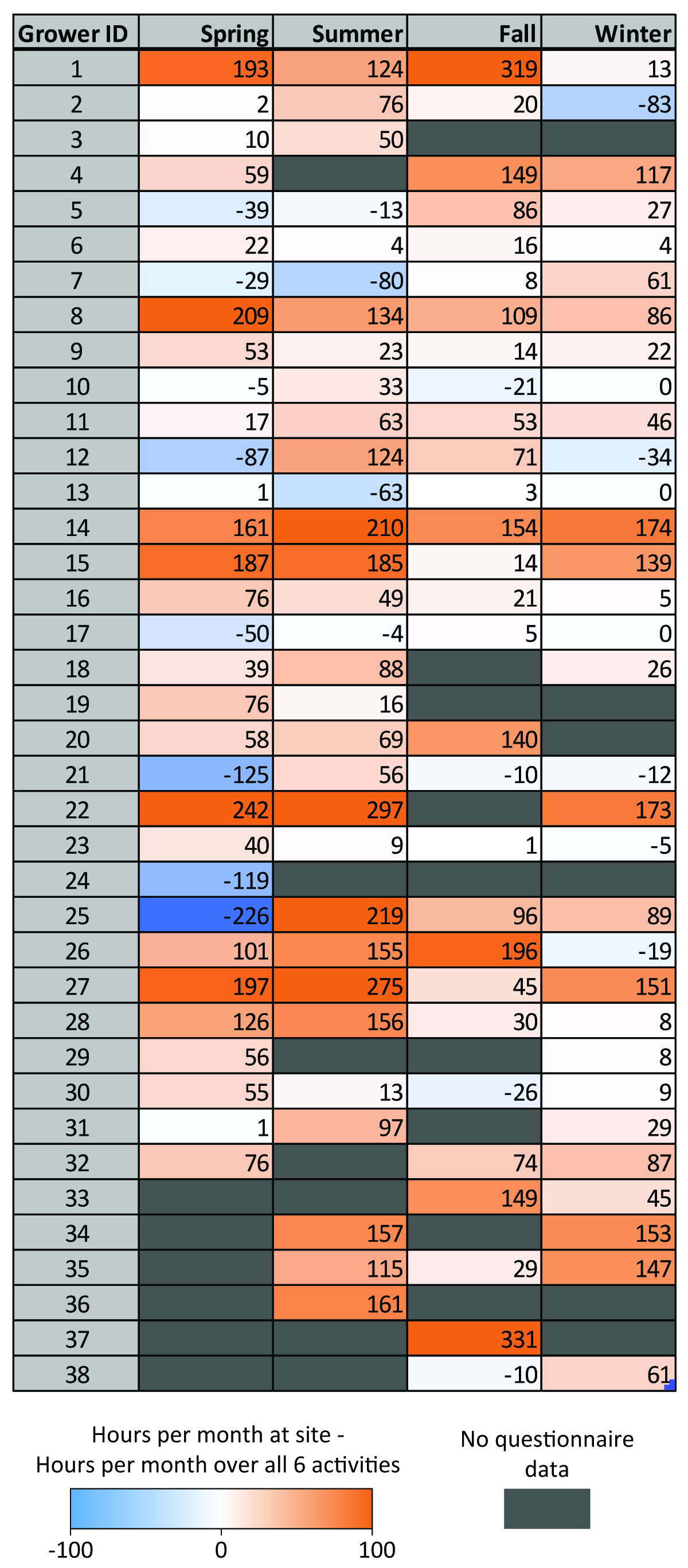


**Figure S3.** Frequency of grower reported exposure reduction behaviors (glove-wearing and handwashing) during/after six meso-activities


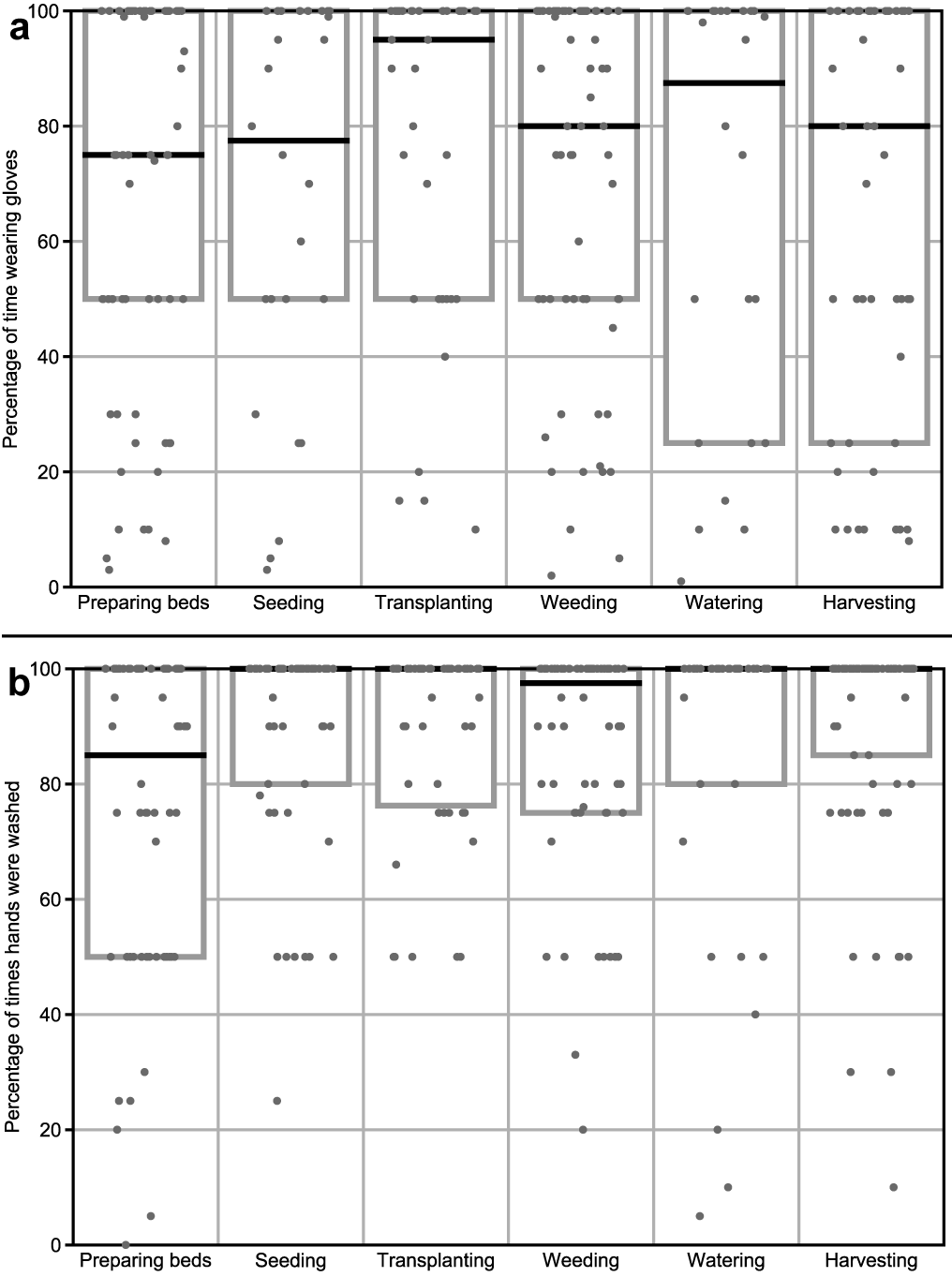


(a) Percentage of time growers wore gloves during each activity. (b) Percentage of times hands were washed immediately after each activity. Boxes represent interquartile ranges (Q1-Q3) and black lines represent medians. Each dot represents one grower’s response for a given season. Results represent only growers who engaged in each activity during a given season.

**Figure S4.** Seasonal average daily doses by soil ingestion method among growers who responded to the soil contact activity questionnaire


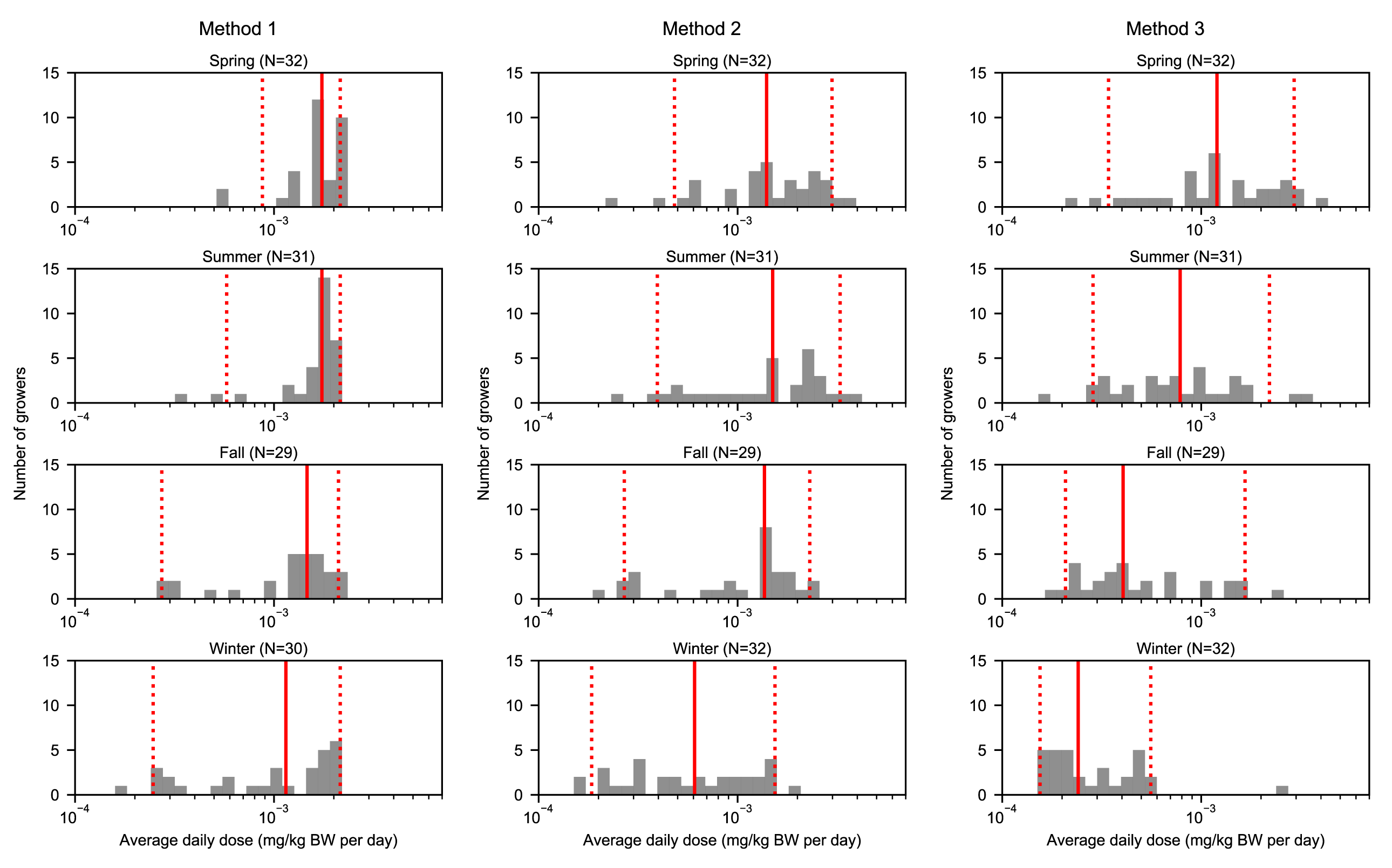


Each histogram shows the distribution of seasonal average daily doses for among the growers who responded to the Soil Contact Activity Questionnaire using one of three types of soil ingestion rates. The left column shows seasonal average daily doses estimated using daily soil ingestion rates (method 1). The center column shows seasonal average daily doses estimated using hourly soil ingestion rates (method 2). The right column shows seasonal average daily doses estimated using hourly-task-specific soil ingestion rates (method 3). The number of growers shown in each histogram is listed above each histogram. The solid red line represents the median, and the dotted red lines represent the 5^th^ and 95^th^ percentiles.

**Figure S5.** Seasonal average daily doses by meso-activity among growers who completed the soil contact activity questionnaire using hourly, task-specific soil ingestion rates.


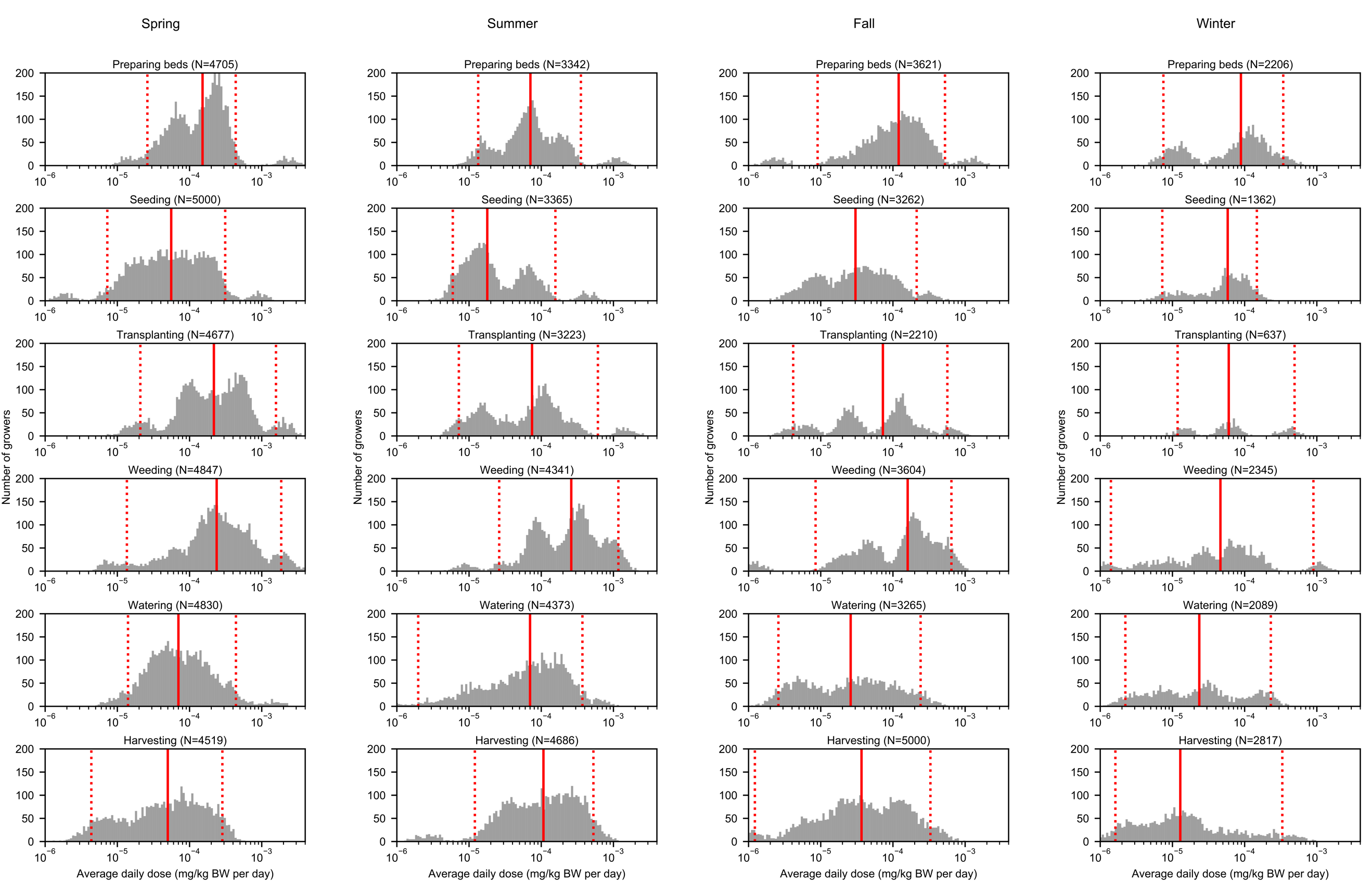


Each histogram shows the distribution of seasonal, task-specific average daily doses for growers who completed the soil contact activity questionnaire simulated growers using hourly-task-specific soil ingestion rates (Method 3). The solid red line represents the median, and the dotted red lines represent the 5^th^ and 95^th^ percentiles.
